# Toxicological Assessment of LN20188, a Botanical Combination of *Withania somnifera* Root and *Abelmoschus esculentus* Fruit Extracts

**DOI:** 10.1155/bmri/6355273

**Published:** 2026-03-31

**Authors:** Ravikumar Madireddy, Sundararaju Dodda, Gopichand Chinta, KrishnaRaju Venkata Alluri

**Affiliations:** ^1^ Department of Toxicology, Laila Nutra Private Limited, R&D Centre, Vijayawada, Andhra Pradesh, India; ^2^ Department of Pharmacology & Medical Affairs, Laila Nutra Private Limited, R&D Centre, Vijayawada, Andhra Pradesh, India

**Keywords:** ashwagandha, genotoxicity, LN20188, no-observed-adverse-effect level (NOAEL), okra, subacute toxicity

## Abstract

LN20188 is a botanical combination composed of extracts derived from *Withania somnifera* (L.) Dunal root and *Abelmoschus esculentus* (L.) Moench fruit. Our earlier investigations revealed the benefits of LN20188 in promoting gut motility and reducing constipation. The current investigation presents the toxicological profile of LN20188 following OECD guidelines for chemical testing. In an acute oral toxicity (AOT) study involving Sprague Dawley rats, no significant signs of toxicity or morbidity were observed, and the lethal dose (LD_50_) was determined to be greater than 2000 mg/kg body weight (b.w). Gross pathological findings further confirmed the safety of LN20188. In a repeated‐dose subchronic toxicological investigation, rats were administered either 500, 1000, or 1500 mg/kg b.w of LN20188 for 90 consecutive days. LN20188 was found to be safe, where data on body weight, food consumption, organ weights, hematology, clinical biochemistry, and biomarkers were comparable to those of vehicle controls. The estimated no‐observed‐adverse‐effect level (NOAEL) for LN20188 in male and female rats is determined to be 1500 mg/kg b.w. Genotoxicity studies, including bacterial reverse mutation, *in vitro*chromosomal aberration (CA), and *in vivo* micronucleus test (MNT) in mouse bone marrow erythrocytes, indicated no clastogenic or mutagenic activity. Results of the bacterial reverse mutation assay showed that LN20188 did not exhibit a notable increase in colony count (mutagenicity) up to 5000 *μ*g/plate. In CA assay, LN20188 concentrations of up to 1000 *μ*g/mL in the short term and 500 *μ*g/mL in continuous exposure did not induce any chromosomal aberrations. Additionally, *in vivo* MNT showed that LN20188, up to 2000 mg/kg b.w, did not elevate the frequency of micronucleated PCEs (MNPCEs). Overall, these studies demonstrate that oral administration of LN20188 does not result in toxicity or genotoxic effects.

## 1. Introduction

Natural products and their bioactive derivatives are known for their medicinal properties and are commonly used in medical treatments worldwide. Throughout human history, plants have been utilized for relieving and treating various ailments affecting not only humans but animals as well, as evidenced by the earliest records [1]. In India′s history, this was no exception, largely due to the presence of one of the world′s most significant biodiversity. The three traditional medical systems, Ayurvedic, Siddha, and Unani, have been practiced for many millennia. Such systems are widely used not just in India but also in Korea, China, Singapore, West Asia, and numerous other nations [2].

*Withania somnifera*, commonly known as ashwagandha, and *Abelmoschus esculentus*, also known as okra, have extensive historical usage in Ayurveda, where they have been valued for their diverse medicinal properties for thousands of years. In Ayurveda, ashwagandha is used to treat various ailments such as inflammatory conditions, insomnia, mood disturbances, loss of physical vigor, and impaired sexual and reproductive health. It also improves cognitive function, immunity, body growth, and cardioprotection. *W. somnifera* has been used as an adaptogen for managing fatigue, stress, and anxiety and for improving overall well‐being [3]. Clinical studies have demonstrated that an individual′s level of stress, anxiety, and serum cortisol is decreased when *W. somnifera* root extract is consumed [4]. Additionally, ashwagandha extracts have demonstrated antibacterial and antidiabetic properties, as well as protection against memory loss and dementia in mice [5–7]. *W. somnifera* contains a diverse range of phytochemicals, including flavonoids, phenolic acids, steroidal alkaloids, saponins, tannins, and withanolides, which are steroidal lactones [8]. The hydroalcoholic extract of *W. somnifera* air‐dried roots standardized to withanolide A by high‐performance thin‐layer chromatography (HPTLC) was found to be nontoxic up to 2000 mg/kg body weight (b.w)/day in a 28‐day repeated‐dose observation in Wistar rats of either sex [9]. Ashwagandha root extract, standardized to contain more than 5% withanolides by high‐performance liquid chromatography (HPLC), demonstrated no chromosomal abnormalities or micronuclei formation in genetic toxicity assessments, including chromosomal aberration (CA) and micronucleus (MN) tests. In acute oral toxicity (AOT) studies, the LD_50_ threshold was determined to be greater than 5000 mg/kg, in accordance with the globally harmonized system (GHS) for classification and labeling of chemical substances and mixtures [10].

*A. esculentus*, or okra, is an edible and polyphenolic‐rich vegetable. Okra is used in traditional medicine to treat inflammation, constipation, hyperlipidemia, microbiological infections, and excessive blood sugar. Okra pods are mucilaginous and contain multiple nutrients, including carotene, folate, B vitamins, vitamin C, amino acids, minerals, carbohydrates, fiber, and unsaturated fatty acids (e.g., oleic and linolenic acids) [11]. Okra is known for its use in treating chronic dysentery, gonorrhea, urinary discharges, bladder blockage, and diarrhea. The fiber content in okra aids in relieving constipation by promoting regular bowel movements [12]. Additionally, okra mucilage contains significant amounts of protein, carbohydrates, neutral sugars, minerals, and complex polysaccharides [13]. In a 28‐day repeated oral toxicological observation, hot water extract of okra polysaccharide is found to be nontoxic up to 1000 mg/kg b.w [14]. Recent preclinical studies have shown okra′s potential for its antifatigue and cognitive‐enhancing effects, as well as effects on gut–brain peptides [15]. It has been demonstrated that okra mucilage lowers blood glucose (Glu) levels [16, 17], normalizes the total cholesterol (TC), and reduces the body weight of obese mice [18].

LN20188 (also referred to as NX18100F4, CL18100F4, or Digexin®) is a proprietary formulation comprising a 1:1 blend of *W. somnifera* root extracts (aqueous and hydroalcoholic) and an aqueous extract of *A. esculentus* fruit. In a recent double‐blind placebo‐controlled clinical study, LN20188 supplementation significantly decreased constipation symptoms and enhanced the quality of life, while it also decreased gastrointestinal (GI) symptoms, serum cortisol, and IL‐6. Furthermore, it improved gastrin, serotonin, and IL‐10 levels, without adversely affecting vital signs or hematological and biochemical parameters [19]. The observations from an earlier placebo‐controlled pilot clinical investigation suggest that LN20188 for 14 consecutive days led to a reduction in constipation and improvement in gastrointestinal function in adults with functional constipation [20]. The objective of the present study is to evaluate the safety of LN20188 by following OECD guidelines. Here, we present a toxicological evaluation of LN20188 that includes AOT and 90‐day repeated‐dose toxicological investigations. For mutagenic assessment of LN20188 bacterial reverse mutation assay, *in vitro* CA tests and *in vivo* micronucleus test (MNT) in mouse bone marrow erythrocytes were performed.

## 2. Materials and Methods

### 2.1. Test Item

LN20188 is a proprietary, patent‐pending formulation composed of an aqueous and hydroalcoholic extract of *W. somnifera* root and an aqueous extract of *A. esculentus* fruit, combined in a 1:1 ratio. The processes for raw material identification, collection, extraction, and HPLC‐based standardization have been described previously [19, 20]. Voucher specimens of these plants are preserved at Laila Nutra Private Limited, R&D Centre, Vijayawada, India, under Accession Numbers CL6852 (*W. somnifera*) and CL6047 (*A. esculentus*).

The composition of LN20188 includes 45% aqueous extract of *A. esculentus* fruit, 45% *W. somnifera* root extract, and 10% excipients. The product is standardized to contain not less than 5% polysaccharides (UV) and 0.2% total withanolides (HPLC). Manufacturing is carried out at a cGMP‐certified facility of Laila Nutra Private Limited, Vijayawada. For the 90‐day repeated‐dose oral toxicity (90D‐Tox) study, formulation homogeneity, concentration, and stability were verified in compliance with good laboratory practice (GLP) to ensure precise dosing. These analyses were not required for acute toxicity or genotoxicity studies, as formulations were freshly prepared and used immediately. Dose adequacy was supported by consistent results and control responses within historical ranges.

### 2.2. Materials and Reagents

*Escherichia coli* (*WP2uvrA*), *Salmonella typhimurium* strains (TA98, TA100, TA1535, and TA1537), and lyophilized rat liver S9 fraction were purchased from Molecular Toxicology Inc. (Boone, North Carolina, United States). DMEM was obtained from Thermo Fisher Scientific (Massachusetts, United States), while fetal calf serum and Bacto agar were sourced from HiMedia Laboratories Pvt. Ltd. (Maharashtra, India) and Becton Dickinson (New Jersey, United States), respectively. The positive controls including 9‐aminoacridine (CAS No. 90‐45‐9), 4‐nitroquinoline‐N‐oxide (CAS No. 56‐57‐5), mitomycin C (CAS No. 50‐07‐7), cyclophosphamide monohydrate (CAS No. 6055‐19‐2), and colchicine (CAS No. 64‐86‐8) were procured from Sigma‐Aldrich (Missouri, United States). All other analytical‐grade reagents were purchased from Sigma‐Aldrich Chemicals (Bengaluru, India). Clinical chemistry and hematology reagents were supplied by Mindray for use with the BS‐240 VET and BC‐500 VET analyzers (Shenzhen, China).

### 2.3. Ethics Approval

All *in vivo* studies, including the AOT, 90D‐Tox, and mammalian bone marrow erythrocyte MNT, were approved by the Institutional Animal Ethics Committee (IAEC) of Laila Nutraceuticals prior to study initiation. The corresponding approval numbers were LN/IAEC/TOX/LN191101 (AOT), LN/IAEC/TOX/LN210507 (90D‐Tox), and LN/IAEC/GTX/LN200901 (MNT).

### 2.4. Animal Care, Housing, and Husbandry

Specific pathogen‐free (SPF) Sprague Dawley rats and Swiss albino mice were procured from Vivo Bio Tech Ltd. (Gajwel, India), Hylasco Biotechnology Pvt. Ltd. (Hyderabad, India), and VAB Biosciences (Hyderabad, India). All animals were housed and handled in accordance with the guidelines of the Committee for the Control and Supervision of Experiments on Animals (CCSEA). Study protocols were approved by the IAEC of Laila Nutraceuticals (Vijayawada, India). Animal rooms were maintained at controlled environmental conditions (22 ± 3^°^C for rats, 30%–70% relative humidity) with a 12‐h light/dark cycle. Sterilized maize cob bedding (Sparconn Life Sciences, Bengaluru, India) was used, and animals had ad libitum access to reverse osmosis (RO) water and pelleted rodent feed. The standard maintenance diet was sourced from Altromin Spezialfutter GmbH & Co. KG (Lage, Germany).

For the AOT study, rats aged 9–10 weeks were obtained from Vivo Biotech Ltd., whereas rats aged 7 weeks used in the 90D‐Tox study were obtained from Hylasco Biotechnology Pvt. Ltd. Swiss albino mice (8–11 weeks) were procured from VAB Biosciences. Following a 1‐week quarantine period, animals were acclimatized for 5–7 days prior to dosing. All experiments were conducted in a GLP‐accredited facility (Laila Nutraceuticals R&D Centre, Vijayawada, India) in compliance with OECD guidelines.

### 2.5. AOT Study

For AOT study, healthy, nulliparous, nonpregnant female Sprague Dawley rats (9–10 weeks, weight: 190–210 g) were used and conducted as per the OECD Test Guideline 425 [21]. Initially, a limit test was carried out on an overnight fasted rat at a dose of 2000 mg/kg b.w p.o. of LN20188. As one rat survived for 48 h, an additional four animals were dosed with the same dose of LN20188. Observations were carried out twice daily to monitor morbidity/mortality and clinical symptoms at 30 min, 1, 2, and 4 h during the initial 24‐h period. Further, animals were closely monitored for changes in the skin, eyes and mucous membrane, body weight, and behavioral patterns for 14 days. On Day 15, following CO_2_ asphyxiation, animals were dissected and observed for gross pathological changes.

### 2.6. Subchronic Toxicological Assessment (90‐Day Repeated‐Dose Study)

A 90D‐Tox study was conducted in compliance with OECD Test Guideline 408 [22] using healthy, 7‐week‐old adult rats. A total of 80 animals (40 males, 227.70–286.39 g, and 40 females, 166.33–203.83 g) were randomized into four groups (10 rats/sex/group). Animals in the vehicle control group (G1) received nanopure water by oral gavage once daily for 90 consecutive days. The low‐ (G2), mid‐ (G3), and high‐dose (G4) groups received LN20188 at doses of 500, 1000, and 1500 mg/kg b.w, respectively, administered once daily by gavage for 90 days.

To assess reversibility, 20 animals (10 males and 10 females) were randomly allocated to two recovery groups (5 rats/sex/group): G1R and G4R. Following the 90‐day dosing period, these animals were maintained for an additional 28 days. The high dose was selected based on mortality observed during the acute toxicity and 14‐day repeated‐dose toxicity studies. The test item was formulated in nanopure water at the required concentrations, and samples were submitted for analytical verification prior to treatment initiation and during Weeks 7 and 13 to confirm concentration and homogeneity. All analytical values were within ±20% of nominal concentrations.

Throughout the study, animals were monitored twice daily for morbidity and mortality, with particular attention to clinical signs of severe pain or distress such as excessive body weight loss, dehydration, self‐injury, or respiratory difficulty. Cage‐side clinical observations were recorded once daily (post‐dose) using individual clinical score sheets, including assessment of body condition, skin and coat, ocular appearance, locomotor activity, autonomic signs, and behavioral changes. Detailed clinical examinations were performed weekly. Body weights were recorded prior to dosing, weekly thereafter, and at necropsy. Rats were housed in groups of 2–3 animals per cage by sex, and food consumption per cage was measured weekly. Ophthalmological examinations were conducted 1 day prior to randomization, during Week 13, and at the end of the recovery phase (Week 17). Neurobehavioral evaluations using a functional observational battery (FOB) were performed during the final week for animals in the main groups.

On Days 91 (main groups) and 119 (recovery groups), animals were fasted overnight and euthanized by CO_2_ asphyxiation, followed by gross necropsy. Absolute weights of major organs (liver, kidneys, heart, brain, spleen, adrenal glands, thyroid with parathyroid, pituitary, thymus, testes, epididymides, prostate, seminal vesicles with coagulating glands, ovaries, and uterus with cervix) were recorded using a precalibrated semimicrobalance (Mettler Toledo, Columbus, Ohio; 0.01 g accuracy).

#### 2.6.1. Hematology and Biochemistry

Clinical pathology evaluations were performed for animals in both the main and recovery (reversal) groups. On Days 91 and 119, animals were fasted overnight prior to blood collection. Approximately 3.5 mL of blood was collected from each anesthetized rat into two vacutainer tubes: one containing K_2_‐EDTA and the other containing a clot activator. Hematological analysis was performed on EDTA‐treated samples using an automated hematology analyzer (BC‐5000 VET, Shenzhen, China). Parameters assessed were a hematology panel consisting of red blood cell (RBC) count, hemoglobin (HGB), hematocrit (HCT), mean corpuscular volume (MCV), mean corpuscular hemoglobin (MCH), mean corpuscular hemoglobin concentration (MCHC), platelet count (PLT), total white blood cell (WBC) count, and differential leukocyte counts evaluated using an automated hematology analyzer (BC‐5000 VET, Shenzhen, China). Reticulocyte counts were assessed manually by smear method, and clotting time was determined using the capillary tube method. A serum clinical chemistry panel including Glu, blood urea nitrogen (BUN), urea (Ur), creatinine (Cre), low‐density lipoprotein (LDL), high‐density lipoprotein (HDL), TC, triglycerides (TG), total bilirubin (T‐bil), aspartate aminotransferase (AST), alanine aminotransferase (ALT), alkaline phosphatase (ALP), total protein (TP), albumin (Alb), calcium (Ca), and phosphorus (Phos) was analyzed using an automated clinical chemistry analyzer (I Lab Aries, United States). Electrolytes, including sodium (Na), potassium (K), and chloride (Cl), were determined using a TurboLyte electrolyte analyzer (CPC Diagnostics Pvt. Ltd., India).

#### 2.6.2. Estrous Cycle Analysis

The estrous cycle stage of female rats in both the main and recovery groups was determined based on vaginal smear cytology by examining cellular patterns on the day of euthanasia (Day 91 for main groups, Day 119 for recovery groups).

#### 2.6.3. Hormone Analysis

Serum thyroid hormone levels, including triiodothyronine (T3), thyroxine (T4), and thyroid‐stimulating hormone (TSH), were quantified using enzyme immunoassay (EIA) kits (Kinesis DX, California, United States) and measured with an automated microplate spectrophotometer (X‐Mark, Bio‐Rad, United States). The analytical sensitivities for T3 (Cat #K11‐0535), T4 (Cat #K11‐0338), and TSH (Cat #K11‐0181) were 125 ng/mL, 0.725 ng/mL, and 0.021 mIU/mL.

#### 2.6.4. Histopathological Evaluation

Collected tissues (adrenal glands, aorta, brain, cecum, colon, duodenum, epididymides, esophagus, eye′s, femur and tibia, skeletal muscle, heart, ileum with Peyer′s patches, jejunum, kidneys, liver, lungs, mesenteric lymph nodes, sciatic nerve, ovaries, pancreas, pituitary, prostate with seminal vesicles and coagulating glands, rectum, salivary glands with mandibular lymph nodes, skin with mammary glands, spinal cord, spleen, sternum, stomach, testes, thymus, thyroid with parathyroid, trachea, urinary bladder, uterus with cervix, and vagina) were fixed and processed for histopathological evaluation, trimmed, and embedded in paraffin. Sections (4 *μ*m) were stained with H&E and examined using a light microscope (Axio Scope, Carl Zeiss, Munich, Germany). Tissues from control and high‐dose groups were evaluated for treatment‐related microscopic lesions.

### 2.7. Genotoxicity Studies

#### 2.7.1. Bacterial Reverse Mutation Assay (Ames Test)

The mutagenic potential of LN20188 was evaluated in accordance with OECD Test Guideline 471 [23], using the plate incorporation method. Mutagenicity of LN20188 was evaluated in *E. coli* (*WP2uvrA*) and *S. typhimurium* tester strains of TA98, TA100, TA1535, and TA1537 in the presence (S9 mix) and absence of a metabolic activation system (phosphate‐buffered saline) [24].

Dimethyl sulfoxide (DMSO) served as the negative control. LN20188 was prepared based on its solubility characteristics. The sensitivity of each bacterial tester strain was verified using spontaneous revertant colony counts obtained from the vehicle and positive control plates, which fell within established historical control ranges. The positive controls induced clear, strain‐specific increases in revertant colonies, thereby confirming the validity of the assay, the responsiveness of the test system to known mutagens, and the functional performance of the metabolic activation (S9) system. Selection of the highest test concentration was guided by a prestudy solubility assessment conducted prior to the dose range–finding experiment. Accordingly, LN20188 was evaluated at 312.5, 625, 1250, 2500, and 5000 *μ*g/plate. Following treatment, plates were maintained at 37°C for 48–72 h. Fresh formulations were prepared on the day of treatment for both the preliminary cytotoxicity assessment and the mutagenicity assay. A stock solution (50 mg/mL) was prepared by weighing the required quantity of LN20188 and dissolving it in the vehicle; subsequent concentrations were generated by serial dilution using the same vehicle. Each concentration was assessed in triplicate, and the mean revertant colony counts were calculated from the three replicate plates. Assay performance was confirmed using appropriate strain‐specific positive controls. In the presence of metabolic activation, 2‐aminoanthracene was used for *S. typhimurium* strains and *E. coli*. In the absence of S9, 9‐aminoacridine (TA1537), sodium azide (TA1535 and TA100), 2‐nitrofluorene (TA98), and 4‐nitroquinoline N‐oxide (WP2uvrA) were used. Positive control solutions were prepared in DMSO, except for sodium azide, which was prepared in nanopure water. A test result was considered positive (mutagenic) when LN20188 produced a reproducible and dose‐related increase in revertant colonies in at least one strain, with or without S9, meeting the predefined fold‐increase criteria (≥ 2‐fold for TA98 and TA100; ≥ 3‐fold for TA1535, TA1537, and *E. coli*) relative to the concurrent negative control. A response was considered negative when no biologically meaningful increase was observed at any tested concentration and revertant counts remained within historical control limits.

#### 2.7.2. In Vitro CA Test

*In vitro* CA test was done in human peripheral blood lymphocyte (PBL) cells as per the OECD Test Guideline 473 [25]. PBLs from a healthy 29‐year‐old male donor (no illness, smoking, viral infection, recent medication, or radiation exposure) were cultured in RPMI 1640 supplemented with 10% FBS and 2% phytohemagglutinin at 37°C in a humidified 5% CO_2_ incubator for 48 hrs [24]. The Institutional Ethics Committee of Alluri Sitarama Raju Academy of Medical Sciences (ASRAM), Eluru, Andhra Pradesh, India, approved the blood withdrawal procedures. In preliminary cytotoxicity assay, concentrations of LN20188 ranging from 6.25 to 2000 *μ*g/mL were prepared by twofold serial dilution. Based on the results of cytotoxicity (significant reduction in mitotic index) of preliminary cytotoxicity assay, the highest dose in CA assay was selected as 1000 *μ*g/mL; the subsequent lower concentration was prepared by twofold serial dilution. Cells were exposed for 4 h with and without metabolic activation for short‐term exposure and 22 h without activation for continuous exposure for completion of 1.5 cell cycle lengths; all the exposures were conducted in duplicate. Cultures were exposed to a metaphase‐arresting agent 2 h before harvesting, later processed for slide preparation with KCL treatment, and cells were fixed and stained with 5% Giemsa stain. In CA assay, blind coded slides are evaluated by scoring 300 cells (in metaphase) per group. Cyclophosphamide and mitomycin C preparations were prepared using the nanopure water which served as positive controls. Evaluation of results was performed in compliance with OECD Test Guideline 473. The test item was considered positive if a statistically significant increase in the incidence of cells with structural CAs, relative to the concurrent negative control, was observed at one or more concentrations, either in the presence or absence of metabolic activation, and the response was reproducible and/or dose dependent. The test item was considered negative when no treatment‐related increase in structural CAs was observed at any concentration.

#### 2.7.3. In Vivo MNT

The*in vivo* MNT was conducted in bone marrow erythrocytes of Swiss albino mice in accordance with OECD Test Guideline 474 [26]. A total of 50 mice (25 males and 25 females, 8–11 weeks old) were randomized into five groups. Distilled water served as the vehicle for preparation of the LN20188 as well as the negative control. Group 1 (G1) received distilled water alone, while Groups 2–4 (G2, G3, and G4) received the LN20188 at doses of 500, 1000, and 2000 mg/kg b.w, respectively, by oral gavage. Two doses of the vehicle or test item were administered at 24‐h intervals. The positive control group (G5) received a single intraperitoneal (ip) injection of cyclophosphamide monohydrate (40 mg/kg b.w) prepared in nanopure water. All animals were observed for clinical signs on the second day and were euthanized approximately 24 h after the final treatment. Bone marrow was collected from both femurs of each animal and flushed using fetal bovine serum (FBS). Following centrifugation, smear slides were prepared from the bone marrow cell pellet, air‐dried, and stained with 5% Giemsa. Slides were examined under a light microscope (ECLIPSE E200, Nikon Corporation, Tokyo, Japan) using a 100× objective. For cytotoxicity assessment, the ratio of polychromatic erythrocytes (PCEs) to normochromatic erythrocytes (NCEs) was determined by scoring 500 total erythrocytes (PCE + NCE) per animal. After confirming cytotoxicity status, slides were blind coded for MN evaluation. For each animal, 4000 PCEs were scored and the frequency of micronucleated polychromatic erythrocytes (MNPCEs) was expressed as a percentage [24]. A statistically significant increase in MNPCE frequency in any treated group relative to the concurrent vehicle control was considered a positive response, whereas the absence of a statistically significant increase was considered negative. The MNPCE frequencies observed in the vehicle control group were within the established laboratory historical control range.

### 2.8. Statistical Analysis

Data were expressed as mean ± SD and analyzed using GraphPad Prism 8.0.2. Normality and variance homogeneity were assessed using Shapiro–Wilk, Kolmogorov–Smirnov, and Bartlett′s tests. Parametric tests (ANOVA with Dunnett′s post hoc, Student′s *t*‐test) were applied when assumptions were met; otherwise, nonparametric tests (Kruskal–Wallis with Dunn′s post hoc, Mann–Whitney *U* test) were used. Ames test results were qualitatively compared with vehicle controls per OECD 471. CA data were analyzed using one‐way ANOVA, as assumptions were satisfied. No trend analysis was performed for MN and CA assays. Pairwise comparisons between treatment and control groups were conducted following OECD 474 and 473 guidelines to assess aneugenic and clastogenic effects.

## 3. Results

### 3.1. AOT Study

In the AOT study, LN20188‐treated animals showed no mortality or treatment‐related clinical signs during the 14‐day observation period. All the animals survived until they were humanely euthanized and showed physiologically normal weight gain during the 14‐day observation. Macroscopic examination at necropsy showed no abnormal gross pathological changes in vital organs, including the brain, heart, lungs, kidneys, and liver, following administration of LN20188 in rats. Based on these findings, the LD_50_ of LN20188 was > 2000 mg/kg b.w. As per the GHS of classification and labeling of chemicals, test items with LD_50_ values between 2000 and 5000 mg/kg are assigned to Category 5 [21].

### 3.2. Subchronic 90D‐Tox Study

The 90‐day subchronic ingestion of LN20188 showed no clinical signs of toxicity or mortality in male and female rats. The animals in both the main and reversal groups are in good health and active. There was no change in ophthalmic parameters or neurobehavioral tests (visual responses, auditory response, gait, and locomotor activity) and grip strength at the end of the trial. Through the trial, no test item–related death or toxicity occurred in any doses of animals.

Table 1 and Figure 1 show the male and female rats′ body weight growth patterns (7 weeks at the start of treatment) at Weeks 4, 8, 12, and 13 (*p* > 0.05) compared to control group animals. A similar pattern was noted by Week 17. No test item–related changes in body weight occurred in either sex across all dose groups over the study period. All groups of LN20188‐treated rats displayed a consistent pattern of body weight gain. Table 2 presents the data of food consumption (grams) in male and female rats. Food consumption data showed there were no substantial (*p* > 0.05) treatment‐related variations among dosage groups (control, 500, 1000, 1500, and reversal) through the study duration.

**Table 1:** Body weights (grams) of the rats during repeated‐dose administration of LN20188 for 90 consecutive days.

Day	LN20188 dose (mg/kg·BW)					
	Main groups				Reversal groups	
	0	500	1000	1500	0	1500
Male						
Predose	256.05 ± 13.78	258.22 ± 14.15	255.66 ± 13.42	252.45 ± 13.02	243.77 ± 7.0	245.05 ± 11.07
4 weeks	424.23 ± 22.18	425.13 ± 21.73	421.79 ± 20.63	420.48 ± 19.18	417.46 ± 14.31	411.66 ± 19.49
8 weeks	497.97 ± 29.53	504.02 ± 23.56	497.6 ± 23.47	497.33 ± 18.18	490.5 ± 17.15	490.54 ± 13.04
12 weeks	531.07 ± 25.59	539.78 ± 23.99	532.58 ± 29.13	530.92 ± 14.29	538.23 ± 27.48	533.11 ± 12.59
13 weeks	538.02 ± 27.06	544.05 ± 26.55	537.89 ± 26.63	537.59 ± 13.56	540.19 ± 24.61	538.58 ± 11.07
17 weeks	—	—	—	—	568.58 ± 29.43	564.89 ± 15.18
Female						
Predose	183.59 ± 7.82	182.3 ± 10.68	181.27 ± 10.11	183.15 ± 9.54	183.89 ± 7.3	183.16 ± 9.41
4 weeks	250.26 ± 12.84	249.54 ± 16.84	247.07 ± 11.44	247.66 ± 14.47	254.59 ± 18.81	254.26 ± 19.27
8 weeks	280.66 ± 19.38	280.89 ± 20.78	279.8 ± 15.15	281.68 ± 11.35	286.71 ± 23.84	286.57 ± 14.42
12 weeks	295.23 ± 23.2	295.9 ± 17.73	294.21 ± 7.82	296 ± 13.12	301.22 ± 28.67	300.05 ± 17.95
13 weeks	297.56 ± 22.64	298.57 ± 17.11	297.38 ± 10.04	298.55 ± 14	304.67 ± 27.54	304.26 ± 17.28
17 weeks	—	—	—	—	322.52 ± 29.02	323.29 ± 20.83

*Note:* Data are presented as mean ± SD in grams. *n* = 20 in main groups (10 males and 10 females) and *n* = 10 in reversal groups (5 males and 5 females).

Figure 1Body weights of (a) male and (b) female rats after repeated‐dose administration of LN20188 for 90 consecutive days.

**Figure:**
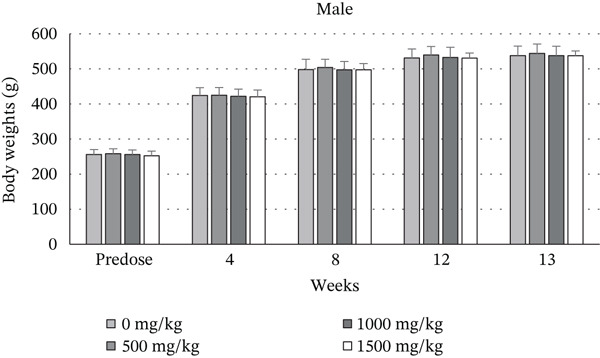
(a)

**Figure:**
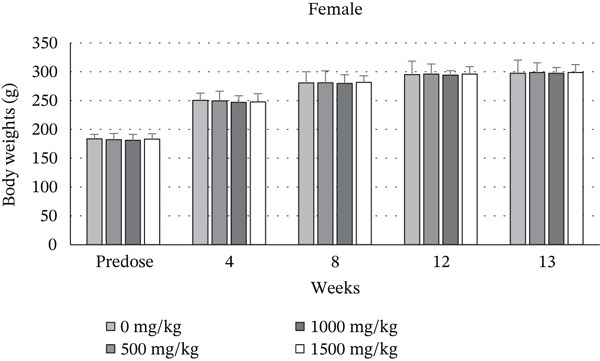
(b)

**Table 2:** Food consumption in grams during the repeated‐dose administration of LN20188 for 90 consecutive days.

Day	LN20188 dose (mg/kg·BW)					
	Main groups				Reversal groups	
	0	500	1000	1500	0	1500
Male						
1–28	198.09 ± 1.44	196.77 ± 4.39	195.80 ± 2.75	195.76 ± 3.35	196.77 ± 2.57	195.96 ± 2.37
29–56	182.65 ± 4.31	182.29 ± 1.03	182.24 ± 2.37	181.75 ± 2.60	183.35 ± 1.55	181.37 ± 0.68
57–90	184.85 ± 4.92	185.68 ± 2.75	184.96 ± 2.63	186.10 ± 3.63	184.47 ± 3.55	185.31 ± 3.21
91–118	—	—	—	—	190.20 ± 0.44	190.56 ± 1.70
Female						
1–28	127.20 ± 3.60	125.50 ± 3.79	123.83 ± 4.26	124.79 ± 2.75	125.08 ± 4.40	126.85 ± 0.34
29–56	107.93 ± 4.56	108.64 ± 3.72	107.01 ± 1.45	105.76 ± 1.04	107.80 ± 5.48	110.05 ± 3.84
57–90	109.67 ± 3.38	110.54 ± 3.42	110.22 ± 0.58	109.26 ± 1.75	109.20 ± 0.72	108.45 ± 1.02
91–118	—	—	—	—	123.51 ± 4.20	122.44 ± 1.46

*Note:* Data are mean ± SD of food consumption in grams. Each main group contains 20 rats (10 males and 10 females), and reversal group contains 10 rats (5 males and 5 females).

Abbreviations: BW, body weight; F, female; M, male.

The hematological profile (Table 3), including HGB concentration, total and differential leukocyte counts, erythrocyte indices (RBC, HCT, MCV, MCH, and MCHC), PLT, reticulocyte count, and clotting time, remained comparable across all groups after 90 days of LN20188 treatment, with no significant intergroup differences.

**Table 3:** Hematological assessments of male and female rats after 90‐day repeated‐dose administration of LN20188.

Parameters	LN20188 dose (mg/kg·BW)					
	Main groups				Reversal groups	
	0	500	1000	1500	0	1500
Male						
WBC (10^3^ cells/*μ*L)	10.9 ± 1.5	10.2 ± 1.2	9.87 ± 1.4	11.08 ± 1.6	8.62 ± 0.5	10.84 ± 2.8
Neutrophils (%)	22 ± 3	25.4 ± 4.2	24.3 ± 4.7	23.3 ± 2.6	23.5 ± 6.8	26.6 ± 3
Lymphocytes (%)	73.9 ± 3.4	70 ± 5.7	69.7 ± 3.8	73.1 ± 3.1	66.7 ± 9.1	64.4 ± 6
Monocytes (%)	3.4 ± 2.4	3.7 ± 2.3	4.7 ± 3.2	2.7 ± 1.3	8.8 ± 3.1	7.8 ± 5.1
Eosinophils (%)	0.8 ± 0.3	1 ± 0.4	1.3 ± 1.4	0.9 ± 0.3	1.1 ± 0.5	1.1 ± 0.5
Basophils (%)	0 ± 0	0 ± 0	0 ± 0	0 ± 0	0 ± 0	0 ± 0
RBC (10^6^ cells/*μ*L)	9.82 ± 0.4	9.32 ± 0.6	9.6 ± 0.5	9.98 ± 0.6	8.67 ± 0.94	9.65 ± 0.2
Hemoglobin (g/dL)	18 ± 0.4	17.5 ± 0.5	17.5 ± 0.6	17.9 ± 0.3	15.8 ± 1.4	16.9 ± 0.6
Hematocrit (%)	52 ± 1.7	50.4 ± 1.7	50.5 ± 2.6	52.4 ± 1.4	46.8 ± 5	49.9 ± 1
MCV (fL)	53 ± 1.4	54.2 ± 3.1	52.6 ± 1.8	52.6 ± 2.5	54 ± 2.6	51.7 ± 1.5
MCH (pg)	18.3 ± 0.6	18.8 ± 1.0	18.2 ± 0.5	18 ± 0.9	18.3 ± 1.0	17.5 ± 0.7
MCHC (g/dL)	34.6 ± 0.6	34.6 ± 0.7	34.7 ± 0.9	34.1 ± 0.5	33.9 ± 1.1	33.7 ± 0.8
Platelets (10^3^ cells/*μ*L)	1066 ± 124	1045 ± 138	984 ± 247	1180 ± 106	909 ± 315	1102 ± 228
Reticulocytes (%)	2.1 ± 0.1	2.1 ± 0.1	2.1 ± 0.1	2.1 ± 0.2	2.1 ± 0.1	2.1 ± 0.1
Clotting time (s)	117 ± 22	144 ± 44	123 ± 30	111 ± 32	108 ± 34	120 ± 21
Female						
WBC (10^3^ cells/*μ*L)	7.34 ± 2.0	8.24 ± 1.6	8.92 ± 2.9	8.11 ± 1.9	9.36 ± 2.2	8.54 ± 1.8
Neutrophils (%)	22.7 ± 3.4	24.6 ± 2.7	22 ± 2.1	22.8 ± 3.3	19.2 ± 1.6	23.8 ± 4.3
Lymphocytes (%)	74 ± 3.4	72 ± 3.2	74.3 ± 2.9	71.9 ± 4.1	76.7 ± 3.0	73.3 ± 5.8
Monocytes (%)	2.5 ± 1.5	2.6 ± 1.2	2.9 ± 1.6	4.3 ± 2.4	3.3 ± 1.6	2.1 ± 1.1
Eosinophils (%)	0.8 ± 0.3	0.8 ± 0.4	0.9 ± 0.3	1 ± 0.3	0.8 ± 0.3	0.9 ± 0.4
Basophils (%)	0 ± 0	0 ± 0	0 ± 0	0 ± 0	0 ± 0	0 ± 0
RBC (10^6^ cells/*μ*L)	8.64 ± 0.3	8.72 ± 0.5	8.8 ± 0.3	8.57 ± 0.5	8.67 ± 0.4	8.61 ± 0.4
Hemoglobin (g/dL)	16.5 ± 0.4	16.7 ± 0.5	16.5 ± 0.4	16.4 ± 0.6	16.4 ± 0.3	16.8 ± 0.5
Hematocrit (%)	47.6 ± 1.1	47.7 ± 2.1	48.2 ± 1.3	47.4 ± 1.7	47.7 ± 1.2	49.6 ± 2.2
MCV (fL)	55.1 ± 1.4	54.7 ± 1.8	54.8 ± 1.7	55.4 ± 2.1	55.1 ± 1.7	57.7 ± 3.3
MCH (pg)	19.1 ± 0.5	19.2 ± 0.7	18.8 ± 0.7	19.2 ± 0.8	19.0 ± 0.8	19.5 ± 0.9
MCHC (g/dL)	34.7 ± 0.5	35 ± 0.8	34.3 ± 0.6	34.6 ± 0.5	34.4 ± 0.5	33.8 ± 0.6
Platelets (10^3^ cells/*μ*L)	1088 ± 136	994 ± 111	1088 ± 149	1093 ± 160	889 ± 295	1116 ± 46
Reticulocytes (%)	2.1 ± 0.1	2.1 ± 0.1	2.1 ± 0.1	2.2 ± 0.2	2 ± 0.1	1.9 ± 0.2
Clotting time (s)	117 ± 22	114 ± 28	120 ± 32	117 ± 33	108 ± 27	96 ± 25

*Note:* Data presented as mean ± SD.

Abbreviations: MCH, mean corpuscular hemoglobin; MCHC, mean corpuscular hemoglobin concentration; MCV, mean corpuscular volume; RBC, red blood cell; WBC, white blood cell.

Table 4 presents serum clinical chemistry findings in male and female rats following administration of LN20188. With the exception of a few parameters, no statistically significant differences were observed between the treated and control groups. In male rats, there are no significant differences in clinical chemistry parameters of main groups compared to animals under no supplementation. Data from the reversal groups also showed a similar trend. In female Sprague‐Dawley (SD) rats, the low‐dose (G2) group demonstrated a significant decrease in LDL (*p* < 0.05) and TG (*p* < 0.001) levels compared to the control group. Similarly, the mid‐dose (G3) group showed a reduction in HDL (*p* < 0.05) and TG (*p* < 0.01) levels. Further, the high‐dose recovery group (G4R) showed an increase in Ur relative to the control group of recovery (G1R) animals. However, these changes did not have any coherence with histological findings as no abnormalities were observed in cardiac tissues when compared with their controls.

**Table 4:** Clinical biochemistry assessment of male and female rats after 90‐day repeated‐dose administration of LN20188.

Parameters	LN20188 dose (mg/kg·BW)					
	Main groups				Reversal groups	
	0	500	1000	1500	0	1500
Male						
Alb (g/dL)	3.8 ± 0.4	3.9 ± 0.5	3.9 ± 0.5	3.8 ± 0.5	3.8 ± 0.2	3.8 ± 0.2
ALP (U/L)	99 ± 26	107 ± 30	109 ± 25	94 ± 21	112 ± 31	92 ± 13
ALT (U/L)	78 ± 19	80 ± 25	68 ± 9	69 ± 14	80 ± 17	73 ± 20
AST (U/L)	140 ± 17	155 ± 30	139 ± 25	132 ± 18	114 ± 17	123 ± 9
T. Bil (mg/dL)	0.2 ± 0	0.1 ± 0.1	0.1 ± 0.1	0.1 ± 0.1	0.3 ± 0.1	0.2 ± 0.1
BUN (mg/dL)	19 ± 3	16 ± 5	15 ± 4	16 ± 2	19 ± 2	17 ± 3
Calcium (mg/dL)	9.1 ± 1.1	9.8 ± 1	8.9 ± 1.2	9.2 ± 1.3	8.9 ± 0.4	9.6 ± 0.6
T. Chol (mg/dL)	81 ± 11	84 ± 8	87 ± 14	85 ± 10	75 ± 15	83 ± 12
Creatinine (mg/dL)	0.53 ± 0.07	0.49 ± 0.09	0.46 ± 0.09	0.49 ± 0.1	0.49 ± 0.05	0.44 ± 0.03
Glucose (mg/dL)	117 ± 18	117 ± 13	114 ± 22	116 ± 13	112 ± 20	96 ± 15
HDL (mg/dL)	48 ± 10	55 ± 10	47 ± 8	47 ± 5	41 ± 4	42 ± 5
LDL (mg/dL)	17 ± 5	19 ± 6	17 ± 4	20 ± 4	21 ± 2	19 ± 2
Phosphorus (mg/dL)	5.5 ± 0.3	5.8 ± 0.4	5.8 ± 0.5	5.5 ± 0.4	5.3 ± 0.3	5.7 ± 0.5
TP (g/dL)	7.9 ± 0.1	7.8 ± 0.4	7.9 ± 0.3	7.7 ± 0.4	7.3 ± 0.7	7.2 ± 0.7
Triglyceride (mg/dL)	78 ± 22	82 ± 20	69 ± 17	71 ± 12	67 ± 10	74 ± 7
Urea (mg/dL)	39 ± 7	35 ± 11	32 ± 8	31 ± 6	39 ± 5	31 ± 5
Sodium (mmol/L)	143.6 ± 1.4	141.8 ± 2.5	143.2 ± 2	142.7 ± 1.2	143.1 ± 1.4	143.1 ± 1.6
Potassium (mmol/L)	5.18 ± 0.32	5.06 ± 0.22	4.99 ± 0.33	4.93 ± 0.22	4.59 ± 0.27	4.74 ± 0.16
Chloride (mmol/L)	101.8 ± 2.1	101.5 ± 4.1	101.2 ± 2.7	100.8 ± 2.5	101.6 ± 2	102.2 ± 1.7
Female						
Alb (g/dL)	3.9 ± 0.5	4.0 ± 0.4	4.1 ± 0.6	3.9 ± 0.5	4.0 ± 0.4	4.2 ± 0.4
ALP (U/L)	57 ± 14	74 ± 24	53.20	61 ± 21	59 ± 19	57 ± 13
ALT (U/L)	59 ± 7	61 ± 8	58 ± 8	54 ± 7	57 ± 11	62 ± 10
AST (U/L)	123 ± 14	120 ± 17	116 ± 19	117 ± 19	114 ± 13	109 ± 14
T. Bil (mg/dL)	0.2 ± 0	0.2 ± 0.1	0.2 ± 0	0.2 ± 0	0.3 ± 0.1	0.3 ± 0.2
BUN (mg/dL)	22 ± 3	20 ± 2	20.2±	19 ± 2	19 ± 3	21 ± 3
Calcium (mg/dL)	9.7 ± 1.2	9.5 ± 0.8	10.5 ± 1.7	9.8 ± 1.2	9.1 ± 0.5	9.4 ± 0.5
T. Chol (mg/dL)	76 ± 10	83 ± 10	78 ± 13	79 ± 11	83 ± 8	81 ± 11
Creatinine (mg/dL)	0.6 ± 0.1	0.59 ± 0.07	0.6 ± 0.13	0.58 ± 0.13	0.49 ± 0.07	0.5 ± 0.04
Glucose (mg/dL)	110 ± 10	107 ± 14	111 ± 8	117 ± 16	98 ± 11	87 ± 8
HDL (mg/dL)	65 ± 15	53 ± 8	49 ± 14^∗^	62 ± 15	58 ± 13	53 ± 13
LDL (mg/dL)	20 ± 3	15 ± 3^∗^	16 ± 4	17 ± 3	22 ± 3	23 ± 5
Phosphorus (mg/dL)	4.4 ± 0.6	4.6 ± 0.5	4.9 ± 0.6	4.9 ± 0.6	4.8 ± 0.9	4.5 ± 1.3
TP (g/dL)	8.6 ± 0.5	8.3 ± 0.5	8.2 ± 0.4	8.1 ± 0.7	7 ± 0.5	7.2 ± 0.5
Triglyceride (mg/dL)	83 ± 11	54 ± 15^∗^	60 ± 17^∗^	72 ± 16	68 ± 16	72 ± 11
Urea (mg/dL)	44 ± 6	42 ± 4	41 ± 4	40 ± 4	40 ± 3	45 ± 6
Sodium (mmol/L)	143 ± 2.4	142.5 ± 1.7	144 ± 1.9	143.3 ± 1.1	142.8 ± 0.2	143.7 ± 1.4
Potassium (mmol/L)	4.91 ± 0.15	4.91 ± 0.18	4.73 ± 0.2	4.8 ± 0.12	4.6 ± 0.31	4.72 ± 0.32
Chloride (mmol/L)	102.7 ± 3.5	101.4 ± 3.1	102.3 ± 2.5	102.5 ± 2.3	102 ± 0.6	103 ± 2

*Note:* Data presented as mean ± SD.

Abbreviations: ALP, alkaline phosphatase; ALT, alanine aminotransferase; AST, aspartate aminotransferase; BUN, blood urea nitrogen; HDL, high‐density lipoprotein; LDL, low‐density lipoprotein; T. Bil, total bilirubin; T. Chol, total cholesterol; TP, total protein.

∗ indicates significant changes (*p* < 0.05) compared with the control group.

After 90 days of treatment, no treatment‐related alterations were observed in serum thyroid hormone parameters (T3, T4, and TSH) in the low‐, mid‐, or high‐dose LN20188 groups compared with the vehicle control group. Similarly, the reversal groups G1R and G4R exhibited no significant changes in thyroid profile (T3, T4, and TSH) (Table 5).

**Table 5:** Hormonal assessments (T3, T4, and TSH) after 90‐day oral administration of LN20188 in male and female rats.

Hormones	Sex	LN20188 dose (mg/kg·BW)					
		Main groups				Reversal groups	
		0	500	1000	1500	0	1500
T3 (ng/mL)	M	0.77 ± 0.15	0.68 ± 0.20	0.64 ± 0.19	0.58 ± 0.17	0.73 ± 0.09	0.80 ± 0.10
	F	0.71 ± 0.11	0.64 ± 0.18	0.75 ± 0.17	0.71 ± 0.16	0.64 ± 0.13	0.72 ± 0.06
T4 (*μ*g/dL)	M	31.38 ± 10.41	34.65 ± 11.44	26.49 ± 10.63	28.29 ± 10.21	31.27 ± 11.75	33.88 ± 10.15
	F	21.74 ± 5.45	25.17 ± 7.90	23.08 ± 8.34	30.08 ± 11.73	25.99 ± 5.30	27.21 ± 6.55
TSH (ng/mL)	M	0.48 ± 0.14	0.58 ± 0.22	0.44 ± 0.07	0.52 ± 0.18	0.46 ± 0.19	0.60 ± 0.18
	F	0.45 ± 0.06	0.52 ± 0.10	0.48 ± 0.09	0.43 ± 0.12	0.58 ± 0.13	0.47 ± 0.11

*Note:* Data presented as mean ± SD. Each main group (*n* = 20; 10 male and 10 female rats); each reversal group (*n* = 10; 5 male and 5 female rats).

Abbreviations: T3, triiodothyronine; T4, thyroxine; TSH, thyroid‐stimulating hormone.

At terminal necropsy, all the organs in study groups appeared normal. The relative and absolute organ weights are comparable with no statistically significant differences except for an increase in the relative organ weight of heart weight observed in female rats. In female rats, the middose group (G3) had a significant increase in relative organ weight of the heart compared to the control (G1). The change was not dose dependent when considering the data of the high‐dose group (G4) (Tables 6 and 7, respectively).

**Table 6:** Absolute and relative organ weights of male rats after 90‐day repeated‐dose administration of LN20188.

Organ	LN20188 dose (mg/kg·BW)					
	Main groups				Reversal groups	
	0	500	1000	1500	0	1500
Liver (g)	14.97 ± 1.50	15.36 ± 1.75	14.65 ± 2.09	15.94 ± 2.09	15.18 ± 0.59	15.82 ± 2.44
% of BW	2.85 ± 0.21	2.89 ± 0.26	2.80 ± 0.34	3.02 ± 0.33	2.74 ± 0.15	2.87 ± 0.45
Kidney (g)	3.25 ± 0.39	3.31 ± 0.34	3.11 ± 0.47	3.11 ± 0.47	3.48 ± 0.15	3.37 ± 0.18
% of BW	0.62 ± 0.07	0.62 ± 0.05	0.59 ± 0.09	0.60 ± 0.008	0.63 ± 0.02	0.61 ± 0.03
Adrenal glands (g)	0.06 ± 0.00	0.06 ± 0.01	0.06 ± 0.01	0.006 ± 0.01	0.07 ± 0.01	0.065 ± 0.01
% of BW	0.01 ± 0.001	0.012 ± 0.001	0.013 ± 0.02	0.12 ± 0.001	0.01 ± 0.001	0.11 ± 0.001
Heart (g)	1.53 ± 0.05	1.62 ± 0.11	1.62 ± 0.09	1.62 ± 0.09	1.57 ± 0.01	1.66 ± 0.05
% of BW	0.29 ± 0.01	0.30 ± 0.02	0.31 ± 0.02	0.31 ± 0.02	0.28 ± 0.01	0.30 ± 0.01
Brain (g)	2.15 ± 0.11	2.18 ± 0.12	2.26 ± 0.15	2.26 ± 0.15	2.21 ± 0.12	2.15 ± 0.04
% of BW	0.41 ± 0.03	0.41 ± 0.02	0.42 ± 0.03	0.42 ± 0.02	0.40 ± 0.03	0.39 ± 0.01
Spleen (g)	0.85 ± 0.11	0.92 ± 0.09	0.91 ± 0.09	0.91 ± 0.09	0.98 ± 0.17	0.92 ± 0.16
% of BW	0.16 ± 0.01	0.17 ± 0.01	0.20 ± 0.08	0.16 ± 0.01	0.17 ± 0.03	0.16 ± 0.03
Thymus (g)	0.40 ± 0.05	0.42 ± 0.04	0.38 ± 0.06	0.38 ± 0.06	0.40 ± 0.05	0.34 ± 0.07
% of BW	0.07 ± 0.01	0.08 ± 0.01	0.08 ± 0.03	0.07 ± 0.009	0.07 ± 0.01	0.06 ± 0.01
Testes (g)	3.55 ± 0.22	3.65 ± 0.11	3.60 ± 0.15	3.60 ± 0.15	3.65 ± 0.24	3.66 ± 0.22
% of BW	0.67 ± 0.04	0.69 ± 0.04	0.62 ± 0.19	0.65 ± 0.02	0.66 ± 0.04	0.66 ± 0.03
Epididymis (g)	1.51 ± 0.16	1.51 ± 0.11	1.47 ± 0.04	1.47 ± 0.04	1.58 ± 0.102	1.58 ± 0.15
% of BW	0.28 ± 0.02	0.28 ± 0.02	0.28 ± 0.01	0.330 ± 0.02	0.28 ± 0.01	0.28 ± 0.02
SV‐CG and prostate gland (g)	3.05 ± 0.66	3.29 ± 0.57	3.42 ± 0.61	3.24 ± 0.62	3.47 ± 0.35	3.36 ± 0.48
% of BW	0.58 ± 0.11	0.62 ± 0.09	0.65 ± 0.10	0.61 ± 0.10	0.62 ± 0.08	0.611 ± 0.01
Thyroid with parathyroid (g)	0.04 ± 0.01	0.04 ± 0.01	0.03 ± 0.01	0.04 ± 0.01	0.04 ± 0.01	0.03 ± 0.01
% of BW	0.01 ± 0.002	0.01 ± 0.002	0.01 ± 0.02	0.01 ± 0.002	0.01 ± 0.002	0.001 ± 0.001
Pituitary gland (g)	0.01 ± 0.001	0.01 ± 0.0002	0.009 ± 0.03	0.009 ± 0.002	0.009 ± 0.002	0.01 ± 0.003
% of BW	0.002 ± 0.0004	0.001 ± 0.0004	0.001 ± 0.006	0.001 ± 0.0004	0.001 ± 0.0005	0.0001 ± 0.0006

*Note:* Data are presented as mean ± SD.

Abbreviations: BW, body weight; SV‐CG, seminal vesicle and coagulating gland.

**Table 7:** Absolute and relative organ weights of female rats after 90‐day repeated‐dose administration of LN20188.

Organ	LN20188 dose (mg/kg·BW)					
	Main groups				Reversal groups	
	0	500	1000	1500	0	1500
Liver (g)	8.16 ± 1.14	7.97 ± 0.73	8.40 ± 0.80	8.31 ± 0.89	8.89 ± 1.03	8.22 ± 1.04
% of BW	2.81 ± 0.26	2.74 ± 0.25	2.88 ± 0.24	2.85 ± 0.19	2.85 ± 0.47	2.59 ± 0.26
Kidney (g)	2.04 ± 0.18	1.92 ± 0.21	1.91 ± 0.21	2.16 ± 0.21	1.98 ± 0.24	1.90 ± 0.14
% of BW	0.71 ± 0.08	0.66 ± 0.07	0.66 ± 0.06	0.74 ± 0.06	0.63 ± 0.11	0.60 ± 0.03
Adrenal glands (g)	0.07 ± 0.01	0.07 ± 0.01	0.06 ± 0.09	0.07 ± 0.07	0.07 ± 0.01	0.07 ± 0.01
% of BW	0.02 ± 0.004	0.025 ± 0.004	0.023 ± 0.0002	0.02 ± 0.0002	0.02 ± 0.003	0.023 ± 0.002
Heart (g)	1.08 ± 0.06	1.70 ± 0.13	1.20 ± 0.11	1.13 ± 0.10	1.13 ± 0.08	1.15 ± 0.09
% of BW	0.37 ± 0.03	0.40 ± 0.03	0.41 ± 0.04^∗^	0.38 ± 0.03	0.36 ± 0.05	0.36 ± 0.03
Brain (g)	2.11 ± 0.11	2.08 ± 0.11	2.07 ± 0.08	1.99 ± 0.12	2.04 ± 0.09	2.04 ± 0.09
% of BW	0.733 ± 0.03	0.71 ± 0.05	0.71 ± 0.03	0.68 ± 0.04	0.65 ± 0.04	0.64 ± 0.04
Spleen (g)	0.16 ± 0.09	0.64 ± 0.05	0.63 ± 0.13	0.66 ± 0.007	0.66 ± 0.09	0.61 ± 0.05
% of BW	0.21 ± 0.02	0.22 ± 0.02	0.21 ± 0.04	0.23 ± 0.02	0.21 ± 0.03	0.19 ± 0.01
Thymus (g)	0.33 ± 0.05	0.30 ± 0.05	0.31 ± 0.05	0.34 ± 0.05	0.28 ± 0.09	0.26 ± 0.03
% of BW	0.11 ± 0.01	0.10 ± 0.01	0.10 ± 0.01	0.11 ± 0.01	0.09 ± 0.03	0.08 ± 0.01
Uterus with cervix (g)	0.83 ± 0.16	0.85 ± 0.14	0.90 ± 0.19	0.77 ± 0.13	0.67 ± 0.07	0.70 ± 0.14
% of BW	0.28 ± 0.04	0.29 ± 0.04	0.31 ± 0.06	0.26 ± 0.04	0.21 ± 0.02	0.22 ± 0.03
Ovaries (g)	0.14 ± 0.02	0.155 ± 0.02	0.14 ± 0.02	0.14 ± 0.02	0.15 ± 0.02	0.15 ± 0.02
% of BW	0.04 ± 0.007	0.053 ± 0.007	0.05 ± 0.008	0.05 ± 0.006	0.048 ± 0.009	0.04 ± 0.00
Thyroid with parathyroid (g)	0.02 ± 0.01	0.023 ± 0.006	0.002 ± 0.005	0.02 ± 0.005	0.02 ± 0.005	0.02 ± 0.004
% of BW	0.01 ± 0.004	0.001 ± 0.002	0.01 ± 0.001	0.01 ± 0.001	0.01 ± 0.001	0.01 ± 0.001
Pituitary gland (g)	0.01 ± 0.002	0.011 ± 0.002	0.01 ± 0.002	0.01 ± 0.003	0.01 ± 0.002	0.009 ± 0.001
% of BW	0.004 ± 0.001	0.004 ± 0.0008	0.003 ± 0.0009	0.0004 ± 0.001	0.003 ± 0.0009	0.003 ± 0.0007

*Note:* Data presented as mean ± SD.

Abbreviation: BW, body weight.

∗ indicates significant changes (*p* < 0.05) compared with the control group.

**Figure 2:**
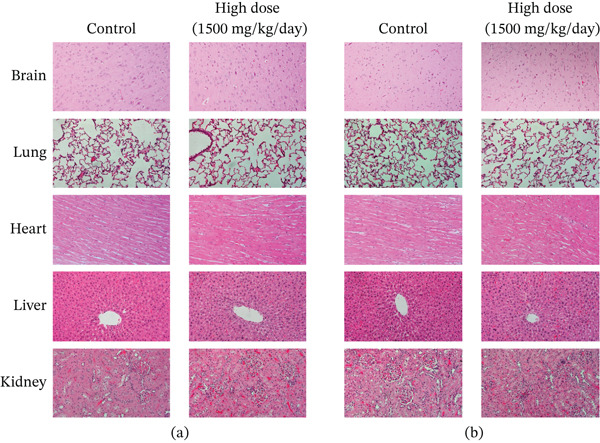
Photomicrographs (200×) of the brain, lung, heart, liver, and kidney of the control and high‐dose (1500 mg/kg b.w) group rats after 90‐day treatment. (a) Male. (b) Female.

**Figure 3:**
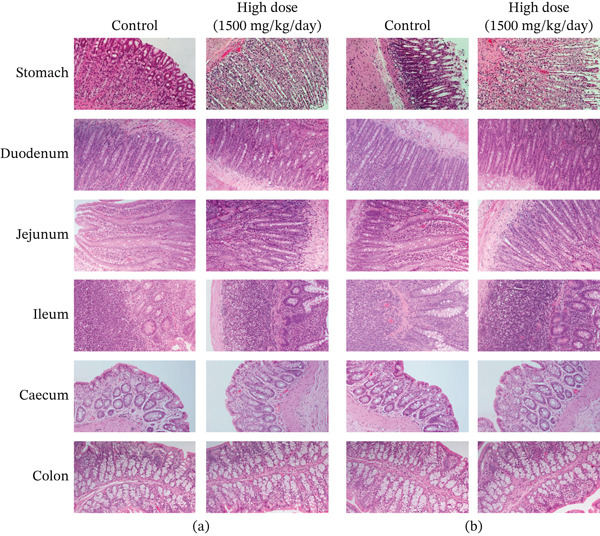
Photomicrographs (200×) of the stomach and small and large intestine tissues of the control and high‐dose (1500 mg/kg b.w) group rats after 90‐day treatment. (a) Male. (b) Female.

Figures 2 and 3 present histological findings on the critical organs of male and female rats. The macro morphology of the vital organs in both male and female rats of the high‐dose group remained unchanged during the LN20188 treatment and reversal period. The main and reverse groups had no treatment‐related effects on essential organs as controls. The macroscopic examination of high‐dose and control group animals revealed no treatment‐related lesions in both sexes till the maximum dose 1500 mg/kg b.w per day. Findings including adrenal vacuolation (high‐dose group), alveolar macrophage infiltration, and reduced pancreatic acinar size (control group) were considered incidental/spontaneous, typical of Sprague Dawley rats, and of no toxicological relevance (Figure 4).

Figure 4(a) Photomicrograph of rat adrenal glands showing diffuse vacuolation zona fasciculata (★). H&E, 200×. (b) Photomicrograph of rat lungs showing multifocal infiltration of alveolar macrophages (**→**). H&E, 200×. (c) Photomicrograph of rat pancreas showing reduced acinar size (▲). H&E, 200×.

**Figure:**
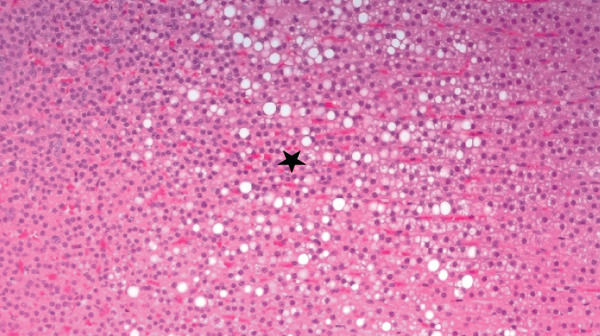
(a)

**Figure:**
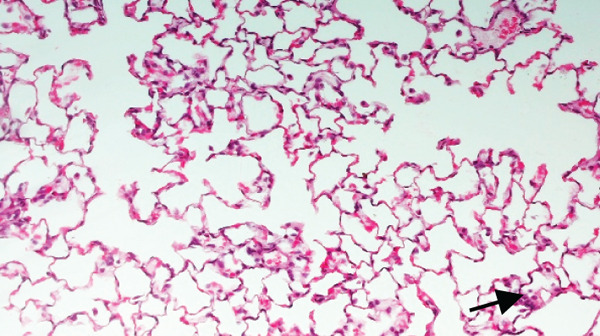
(b)

**Figure:**
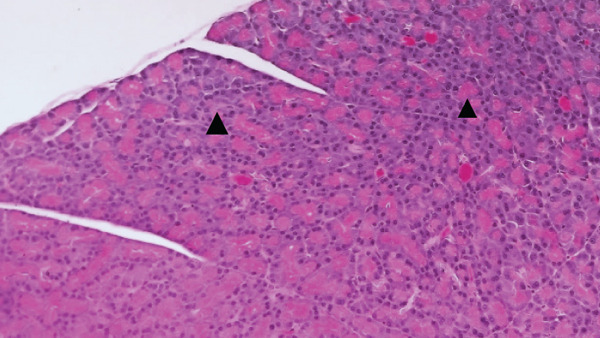
(c)

### 3.3. Bacterial Reverse Mutation Assay (Ames Test)

LN20188 did not exhibit a fold increase in colony count up to 5000 *μ*g/plate when evaluated on five distinct strains of *S. typhimurium* and *E. coli* WP2uvrA, both in the absence and presence of metabolic activation as it met the criteria for negative response (Table 8). Further, there was no visible precipitation observed on the plates. No cytotoxic effects (background lawn reduction or decreased revertant counts) were noted at any concentration, with or without metabolic activation (5% *v*/*v* S9), relative to the vehicle control.

**Table 8:** Observations of Ames test in the presence and absence of metabolic activation.

S9	Treatments	Conc. (*μ*g/plate)	Revertant colony count (*m* *e* *a* *n* ± *S* *D*)				
			TA1537	TA1535	TA98	TA100	WP2uvrA
+S9	DMSO^a^	0	11.00 ± 3.00	13.00 ± 1.00	29.67 ± 2.52	111.00 ± 8.54	14.00 ± 3.00
	LN20188	312.5	10.33 ± 2.08 (0.94)	12.33 ± 0.58 (0.95)	30.33 ± 2.08 (1.02)	121.00 ± 3.00 (1.09)	13.33 ± 1.53 (0.95)
		625	11.00 ± 1.00 (1.00)	12.00 ± 2.65 (0.92)	27.33 ± 3.51 (0.92)	111.00 ± 3.00 (1.00)	11.67 ± 2.08 (0.83)
		1250	10.67 ± 1.53 (0.97)	14.33 ± 1.53 (1.10)	28.33 ± 2.52 (0.96)	126.33 ± 5.03 (1.14)	14.33 ± 2.08 (1.02)
		2500	8.67 ± 1.53 (0.79)	12.00 ± 2.00 (0.92)	28.67 ± 2.52 (0.97)	128.67 ± 3.06 (1.16)	12.00 ± 1.00 (0.86)
		5000	8.33 ± 0.58 (0.76)	12.33 ± 1.53 (0.95)	26.00 ± 2.00 (0.88)	128.33 ± 6.03 (1.16)	12.33 ± 1.53 (0.88)
	2‐Amino‐anthracene^b^	20	194.00 ± 9.17 (17.64)	1020.67 ± 58.80 (78.15)	1172.00 ± 33.41 (39.51)	1230.00 ± 46.13 (11.08)	—
		30	—	—	—	—	210.67 ± 23.44 (15.05)
−S9	DMSO^a^	0	10.33 ± 1.53	12.00 ± 2.00	30.00 ± 1.00	127.00 ± 5.57	12.67 ± 1.53
	LN20188	312.5	11.33 ± 3.06 (1.10)	11.33 ± 1.53 (0.94)	26.67 ± 1.53 (0.89)	124.67 ± 6.03 (0.98)	11.00 ± 1.00 (0.87)
		625	10.33 ± 2.31 (1.00)	13.67 ± 1.53 (1.14)	28.67 ± 2.52 (0.96)	132.33 ± 3.51 (1.04)	13.00 ± 1.00 (1.03)
		1250	10.00 ± 1.00 (0.97)	13.00 ± 2.00 (1.08)	26.33 ± 2.52 (0.88)	114.00 ± 5.00 (0.90)	10.67 ± 0.58 (0.84)
		2500	9.33 ± 1.15 (0.90)	11.00 ± 1.00 (0.92)	27.67 ± 3.06 (0.92)	122.33 ± 6.51 (0.96)	12.00 ± 1.00 (0.95)
		5000	10.33 ± 2.08 (1.00)	12.67 ± 2.08 (1.06)	28.33 ± 3.06 (0.94)	125.67 ± 6.11 (0.99)	12.00 ± 2.00 (0.95)
	9‐Aminoacridine^b^	50	195.00 ± 14.73 (18.87)	—	—	—	—
	Sodium azide^b^	10	—	1087.33 ± 54.78 (90.61)	—	1309.33 ± 41.97 (10.31)	—
	2‐Nitrofluorene^b^	25	—	—	1279.33 ± 38.18 (42.64)	—	—
	4‐NQO^b^	3	—	—	—	—	227.67 ± 9.61 (17.97)

*Note:* Data are presented as mean ± SD (mutation rate).

Abbreviations: DMSO, dimethyl sulfoxide; NQO, nitroquinoline oxide.

^a^Vehicle controls.

^b^Positive controls.

### 3.4. In Vitro CA Test

Under short‐term exposure, cultures treated with 250, 500, and 1000 *μ*g/mL of the test item showed mitotic index reductions of 1.71%, 9.41%, and 25.58%, respectively, without metabolic activation, and 8.19%, 12.63%, and 22.21%, respectively, with metabolic activation. In continuous exposure without metabolic activation, treatments at 125, 250, and 500 *μ*g/mL resulted in mitotic index reductions of 15.00%, 17.88%, and 26.16%, respectively.

LN20188 showed no clastogenic effects under study conditions. Negative controls matched historical data, while positive controls confirmed assay validity. No dose‐related increases in CAs, polyploidy, or endoreduplication were seen. Cytotoxicity, as measured by mitotic index, showed acceptable reductions at the highest concentrations tested, supporting the selection of dose levels. Overall, all test results were within the historical negative control range, indicating that LN20188 is non‐clastogenic under the conditions of this study (Table 9).

**Table 9:** Observations on chromosomal aberration (CA) induced by LN20188.

Treatments	Conc. (*μ*g/mL)	Number of cells scored	Mitotic index			Number of cells scored	Number of structural aberrant cells without gap		
			−S9		+S9		−S9		+S9
			4 h	22 h	4 h		4 h	22 h	4 h
DMSO^a^	0	1000	—	—	—	300	1	1	1
	125	1000	—	15.0	—	300	—	1	—
	250	1000	1.71	17.88	8.19	300	1	0	1
	500	1000	9.41	26.16	12.63	300	1	1	1
	1000	1000	25.58	—	22.21	300	1	—	1
Mitomycin C^b^	0.3	1000	24.04	31.26	—	300	9 ^∗^	9 ^∗^	—
Cyclophosphamide^b^	10	1000	—	—	27.94	300	—	—	8 ^∗^

^a^Vehicle control (DMSO, dimethyl sulfoxide).

^b^Positive controls (mitomycin C and cyclophosphamide monohydrate).

∗ indicates significant changes (*p* < 0.05) compared with vehicle control.

### 3.5. In Vivo MNT

No mortality or test item–related adverse clinical signs were observed during the 48‐h observation period following LN20188 administration at doses up to 2000 mg/kg b.w. There were no statistically significant changes in the PCE/TE ratio and no evidence of a treatment‐related, dose‐dependent increase in MNPCE frequency. All observations were within the established laboratory historical negative control range, and concurrent negative control values were comparable to historical control data. The positive control, cyclophosphamide induced the expected statistically significant increase in MNPCE frequency within historical limits, confirming the adequacy and validity of the test system (Table 10).

**Table 10:** Micronucleated polychromatic erythrocytes (MNPCEs) in LN20188‐treated mouse bone marrow.

Treatments	Dose (mg/kg·BW)	Number of mice	PCE/TE	% reduction	% MNPCE
Vehicle control^a^ LN20188	0	10	0.510	—	0.03 ± 0.02
	500	10	0.502	1.5	0.03 ± 0.02
	1000	10	0.500	1.9	0.03 ± 0.03
	2000	10	0.503	1.3	0.03 ± 0.03
Cyclophosphamide monohydrate^b^	40	10	0.505	0.9	1.51 ± 0.26 ^∗^

*Note:* Each group contains 10 animals (5 male and 5 female).

Abbreviations: BW, body weight; MNPCE, micronucleated polychromatic erythrocyte; PCE, polychromatic erythrocyte; TEs, total erythrocytes.

^a^Distilled water.

^b^Positive control.

∗ indicates significant changes (*p* < 0.05) compared with vehicle control.

## 4. Discussion

This study presents the toxicological investigations of LN20188, a nutraceutical composition comprising extracts from the roots of *W. somnifera* (L.) Dunal (ashwagandha) and the fruits of *A. esculentus* (L.) Moench (okra). Ashwagandha and okra have been integral to Ayurvedic medicine for thousands of years, revered for their diverse therapeutic properties. The edible pods of okra are consumed as a vegetable and are often referred to as a perfect villager’s vegetable [27]. Ashwagandha is also incorporated into traditional food items such as Namakpara and Missi roti [28]. These plant materials are well known for their potent pharmacological properties and diverse phytochemical profiles, frequently used in traditional and alternative medicine. The growing consumption of nutraceuticals, especially at elevated doses and over prolonged periods, may lead to adverse health effects. Since these products are not subject to rigorous preclinical testing for efficacy and toxicity by regulatory authorities, the possibility of negative impacts on consumer health remains a concern [29]. This necessity aligns with the recommendations of the European Food Safety Authority (EFSA) and the United States Food and Drug Administration (US‐FDA) [30, 31]. In this context, the present study investigates the toxicological impact of LN20188 per OECD test guidelines.

In the current investigation, the AOT study in Sprague Dawley rats showed that the LD_50_ of LN20188 is greater than 2000 mg/kg b.w. LN20188 did not exhibit any acute toxicity, mortality, morbidity, abnormal clinical signs, or treatment‐related gross pathological findings. An additional upper fixed‐dose level of 5000 mg/kg was deemed unnecessary, as 2000 mg/kg b.w exhibited no toxicity, in accordance with OECD 425 guidelines [21]. Expanding on the acute toxicity study, an oral subchronic toxicity investigation was conducted using Wistar rats. LN20188 was administered once daily for 90 consecutive days. In line with OECD guidelines [22], this study seeks to detect potential toxic effects from repeated oral exposure to test materials over a defined portion of an animal′s lifespan, limited to a maximum of 10% of its total duration. In the subchronic toxicity study, oral administration of LN20188 at doses up to 1500 mg/kg b.w did not produce any treatment‐related changes in clinical signs, behavior, body weight, or food consumption in either male or female rats from the main and recovery (reversal) groups compared with controls. If a test substance is toxic, it typically impacts food intake and animal growth [32]. In this subchronic study, no statistically significant differences in body weights were observed. LN20188‐treated animals did not show any abnormalities during the FOB assessments, sensory reactivity, grip strength, or locomotor activity [33]. These results indicated that oral administration of LN20188 did not lead to significant changes in growth, food intake, or neurotoxic effects in rats.

No statistically significant treatment‐related alterations were observed in hematological parameters in male or female rats administered with LN20188 compared with the control group. Although minor variations were noted in a few parameters, these changes were within normal physiological ranges and were considered consistent with age and sex‐related biological variability. Overall, the findings indicate that LN20188 did not produce hematological toxicity under the conditions of this study.

In clinical biochemistry analysis, assessing the toxicity of test items on liver and kidney function is essential [34]. In this study, female rats in the low‐dose group (G2) exhibited significantly reduced LDL and TG levels, and the mid‐dose (G3) group showed a decrease in HDL compared to control counterparts. Male animals in the high‐dose recovery group (G4R) showed lower Ur levels compared to control. Thyroid hormone analysis data indicate that no treatment‐related changes were observed in either male or female rats across both the control and supplement groups. Furthermore, the recovery groups did not exhibit any significant differences. During gross pathology examination, no significant alterations were noted in LN20188‐treated animals. Histopathological evaluation of sampled organs from LN20188‐treated animals revealed a few spontaneous lesions unrelated to treatment. Hence, these biochemical changes were considered as transient and not related to treatment. The current investigation is limited by the absence of an assessment of parameters associated with herb‐induced liver injury, specifically the Roussel Uclaf Causality Assessment Method (RUCAM) scores. Although ashwagandha is a component of LN20188, we do not expect any elevation in RUCAM scores. This expectation is supported by previous reports indicating that increased RUCAM scores were primarily observed in individuals with pre‐existing liver conditions who consumed generic ashwagandha formulations [35]. Based on the outcomes of the study evaluations, the dose of 1500 mg/kg b.w was determined as the no‐observed‐adverse‐effect level (NOAEL).

Results from the bacterial reverse mutation assay (Ames test) indicated LN20188 is nonmutagenic, as evidenced by the absence of significant or dose‐dependent increases in revertant counts. In both *in vitro* CA and *in vivo* MNTs, LN20188 did not show statistically significant increases in aberrations or MNPCE numbers compared to control. Overall, findings from *in vitro and in vivo* genotoxicity conducted under the applied test conditions indicate that LN20188 does not exhibit mutagenic or clastogenic potential, indicating no evidence of DNA or chromosomal damage.

## 5. Conclusions

Based on the findings, LN20188 (NX18100F4 or CL18100F4 or Digexin®), a blend of extracts from *W. somnifera* (L.) Dunal root and *A. esculentus* (L.) Moench fruit, demonstrates a robust safety profile. No systemic toxicological or genotoxic effects were observed following oral administration of LN20188. LN20188 administration for 90 consecutive days showed no abnormal findings in clinical, laboratory, macroscopic, or microscopic examinations, with no observed effects at doses up to 1500 mg/kg body weight. The no‐observed‐adverse‐effect level (NOAEL) for LN20188 in both male and female rats was determined to be 1500 mg/kg b.w. These results confirm that oral administration of LN20188 does not induce systemic or genotoxic effects.

**Nomenclature**
ALP: alkaline phosphatase
ALT: alanine aminotransferase
ANOVA: analysis of variance
AOT: acute oral toxicity
AST: aspartate aminotransferase
BSA: body surface area
BUN: blood urea nitrogen
b.w: body weight
CC: cubic centimeters
CMC‐Na: sodium carboxymethyl cellulose
CO_2_
: carbon dioxide
CCSEA: Committee for the Control and Supervision of Experiments on Animals
CT: clotting time
DMEM: Dulbecco′s modified Eagle medium
DNA: deoxyribonucleic acid
EFSA: European Food Safety Authority
EIA: enzyme immunoassay
EOSI%: relative eosinophil count
FBS: fetal bovine serum
FOB: battery of functional observation
GHS: globally harmonized system of classification and labeling of chemicals
GLP: good laboratory practice
H&E: hematoxylin and eosin
HCl: hydrochloric acid
HCT: hematocrit
HDL: high‐density lipoprotein
HGB: hemoglobin
HPLC: high‐performance liquid chromatography
IAEC: Institutional Animal Ethics Committee
LD_50_
: median lethal dose
LDL: low‐density lipoprotein
LYMP%: relative lymphocyte count
MCH: mean corpuscular hemoglobin
MCHC: mean corpuscular hemoglobin concentration
MCV: mean corpuscular volume
MMTS: maximum mean total score
MNPCEs: micronucleated polychromatic erythrocytes
MONO%: relative monocyte count
NEUT%: relative neutrophil count
NOAEL: no‐observed‐adverse‐effect level
OECD: Organization for Economic Cooperation and Development
PCEs: polychromatic erythrocytes
RBCs: red blood cells
RO: reverse osmosis
SPF: specific pathogen‐free
T‐bil: total bilirubin
T. Chol: total cholesterol
T3: triiodothyronine
T4: thyroxine
TG: triglycerides
TP: total protein
TSH: thyroid‐stimulating hormone
Ur: urea
WBCs: white blood cells

## Author Contributions

**Ravikumar Madireddy:** study director, investigation, resources, formal analysis, data curation. **Sundararaju Dodda:** methodology, validation, data curation, data analysis, writing—review and editing, visualization. **KrishnaRaju Venkata Alluri:** writing—review and editing, study supervision, funding acquisition, conceptualization. **Gopichand Chinta:** writing—original draft, review and editing, software, visualization, data analysis, proofreading.

## Funding

The study is supported by Laila Nutra Private Limited (LN/20188/18).

## Conflicts of Interest

The authors are employees of Laila Nutra Private Limited, R&D Centre, Vijayawada, India, and have conflicts of interest for the research, authorship, and publication of this article.

## Supporting information


**Supporting Information** Additional supporting information can be found online in the Supporting Information section. Table S1. Effect of 90‐day oral administration of LN20188 on body weights of male and female rats of reversal groups.

## Data Availability

Data are available on request from the authors.
