# Supplementary material for: Toxicological Assessment of LN20188, a Botanical Combination of Withania somnifera Root and Abelmoschus esculentus Fruit Extracts
Source: Biomed Res Int. 2026 Mar 31;2026:6355273. doi: 10.1155/bmri/6355273 (PMC13140333; doi:10.1155/bmri/6355273)
Supplement: Supplementary file 1 — Supporting Information Additional supporting information can be found online in the Supporting Information section. Table S1. Effect of 90‐day oral administration of LN20188 on body weights of male and female rats of reversal groups. [file BMRI-2026-6355273-s001.docx]

**Table S1: Effect of 90-day oral administration of LN20188 on body weights of male and female rats**

| **Days** | **LN20188 dose (mg/kg.BW)** | |
| --- | --- | --- |
|  | **Reversal groups** | |
|  | **0mg** | **1500mg** |
| Male | | |
| Predose | 243.77±7.0 | 245.05±11.07 |
| 4 weeks | 417.46±14.31 | 411.66±19.49 |
| 8 weeks | 490.5±17.15 | 490.54±13.04 |
| 12 weeks | 538.23±27.48 | 533.11±12.59 |
| 13 weeks | 240.19±24.61 | 538.58±11.07 |
| 17 weeks | 568.58±29.43 | 564.89±15.18 |
| Female | | |
| Predose | 183.89±7.3 | 183.16±9.41 |
| 4 weeks | 254.59±18.81 | 254.26±19.27 |
| 8 weeks | 286.71±23.84 | 286.57±14.42 |
| 12 weeks | 301.22±28.67 | 300.05±17.95 |
| 13 weeks | 304.67±27.54 | 304.26±17.28 |
| 17 weeks | 322.52±29.02 | 323.29±20.83 |

Data presented as mean ± SD of body weight (BW) in g. n=20 in main groups (10 males and 10 females) and n=10 in reversal groups (5 males and 55 females).
